# Prevalence of Refractive Errors Among School-Going Children in Urban Versus Rural Areas

**DOI:** 10.7759/cureus.59197

**Published:** 2024-04-28

**Authors:** Tanmay Srivastava, Aalok Kumar, Ekagrata Shukla, Vibha Singh, Lavanya Anuranjani

**Affiliations:** 1 Ophthalmology, Maa Vindhyavasini Autonomous State Medical College, Mirzapur, IND; 2 Ophthalmology, Institute of Medical Sciences, Banaras Hindu University, Varanasi, IND; 3 Obstetrics and Gynecology, Heritage Institute of Medical Sciences, Bhadawar, IND

**Keywords:** myopia, electronic devices, prevalence, school-going children, refractive error

## Abstract

Background: The most common cause of visual impairment globally is refractive error. The prevalence of refractive error has been on the rise since the coronavirus disease 2019 (COVID-19) pandemic, possibly due to increased indoor activities and excessive use of electronic devices. Impaired vision during childhood can affect the overall development of a child adversely, and it often remains unreported due to the inability of children to complain about impaired vision.

Aim: The main aim of this study was to assess the prevalence of refractive errors among school-going children in urban and rural areas.

Methods: This was a cross-sectional study that included 2024 children going to schools situated in urban and rural areas. All study subjects were tested for visual acuity for distance using Snellen’s chart with and without glasses after taking a brief history regarding visual complaints. All children who had visual acuity less than 6/6 on Snellen’s chart underwent refraction check-ups. Retinoscopy was performed in all study subjects. Analysis of the collected data was done using SPSS for Windows, Version 16.0 (Released 2007; SPSS Inc., Chicago, United States). The analysis of numerical data was done by an unpaired t-test, and the analysis of categorical data was done by a chi-square test. A P-value of less than 0.05 was considered statistically significant.

Results: The mean age of the children was 10.92 ± 2.73 years, with 10.93 ± 2.73 years in urban and 10.91 ± 2.73 years in rural groups. Females (n=1031; 50.93%) were more in number than males (n=993; 49.06%). The overall prevalence of refractive error was 17.43%. The prevalence was higher in urban areas (22.14%) than in rural areas (12.71%). The age group below 10 years comprised 886 (43.77%) study subjects, and 218 (62.1%) children with refractive error had no ocular complaints. The most common refractive error found in this study was simple myopia in both groups, and the least common was astigmatism. The prevalence of uncorrected refractive error was higher in urban school-going children as compared to rural children.

Conclusion: The prevalence of refractive error was 17.43% in our study. The prevalence was high in urban areas (22.67%) as compared to rural areas (13.12%). Regular screening of school-going children for refractive errors should be done. Also, awareness regarding the use of electronic gadgets must be raised, especially among urban children.

## Introduction

Refractive error (ametropia) is a condition in which incident parallel rays of light are not able to converge at a sharp focus on the retina with accommodation at rest. Refractive errors include myopia (shortsightedness), hypermetropia (longsightedness), and astigmatism [1]. Refractive error is caused by a mismatch between the combined refractive power of the cornea and lens of the eye and the axial length of the eyeball. This usually occurs during childhood, when the eye is growing. It is known that both hereditary and environmental factors also influence the development of refractive error. The exact causes of refractive error are still under study [1].

The most common cause of visual impairment around the world is uncorrected refractive errors [2]. In children, uncorrected refractive error has a profound effect on their overall development, and most importantly, its consequences on educational and psychosocial development are of extreme concern [3,4]. In 1994, eye screening programs at schools were started by the Government of India under the National Program for Control of Blindness [5]. In 1999, WHO launched Vision 2020: Right to Sight to eliminate avoidable blindness like refractive errors, cataracts, xerophthalmia, trachoma, and other causes of childhood blindness by the end of 2020. Refractive errors (mostly myopia) are more common nowadays and are on the rise globally [6].

School-going children generally are not aware of their defective vision, and they mostly have no complaints regarding their vision. Sometimes they adjust to their visual problems by either changing their position in the classroom, moving closer to the objects, or avoiding the tasks deliberately, which requires more visual attention. Screening of children is always recommended for early detection of refractive errors among them so that timely intervention can provide them with the best visual outcomes leading to better learning [7]. Hence, it is necessary to estimate the prevalence both at the community and at the school level to aid in the planning and implementation of refractive error services for children. Region-specific prevalence estimates are necessary for policy decisions and the evidence-based allocation of resources.

In the available literature, there are several studies with a focus on the prevalence of refractive errors worldwide, including in India [8-12]. However, not many studies on the necessity of early screening have been conducted in northern India with sample sizes of more than 2000. The aim of our study was to study the prevalence of refractive errors in school-going children and assess any change in prevalence after coronavirus disease 2019 (COVID-19), as the use of electronic gadgets and online classes has increased since then. It is important to note that most children with refractive errors have no symptoms and go undetected, which leads to anisometropic amblyopia. Hence, this study will help to create awareness and emphasize the need for and importance of early detection and timely management of refractive errors.

## Materials and methods

It is a cross-sectional, observational study conducted at rural and urban schools within a 15-kilometer radius of the Institute of Medical Sciences, Banaras Hindu University, Varanasi, India, for a period of 22 months from December 2021 to October 2023. The schools that came under Varanasi Municipal Corporation were identified as urban schools and the remaining schools in peripheral areas were identified as rural. The study was approved by the Institutional Ethical Committee, Institute of Medical Sciences, Banaras Hindu University (approval number: Dean/2021/EC/2630). Written informed consent was obtained from the parents or guardians of the children.

The study included school-going children aged 6-15 years who were able to follow instructions and children with developmental disorders and ocular pathology, and whose parents or guardians did not give consent to participate in the study were excluded.

Sample size

Sample size was calculated using the formula n = [DEFF*Np (1-p)/[d2/Z21-a*(N-1) +p*(1-p)].

Where N = population size, p= prevalence, d = precision, DEFF = design effect.

The sample size came out to be 2024. Four schools were randomly selected each from the rural as well as from the urban area. About 250 randomly selected children from each school who met the inclusion and exclusion criteria were included in the study.

Procedure

All the included students underwent distant visual acuity testing using Snellen’s visual acuity chart. If the presenting maximum vision was <6/6 on Snellen’s chart, the child was considered for refractive error testing. Following this, a retinoscopy under cycloplegics was performed on all the children.

Statistical analysis

SPSS for Windows, Version 16.0 (Released 2007; SPSS Inc., Chicago, United States) was used for the analysis of the data. The numerical and continuous data were expressed as mean ± standard deviation, and the categorical data were expressed as percentages. Unpaired t-test was used to analyze the numerical and continuous data. The categorical data was analyzed using chi-square test. Fischer's exact test was used when more than 20% of the cells had a value less than 5. Bar charts and pie diagrams were used for the presentation of the data. A p-value of less than 0.05 was considered statistically significant.

## Results

This study comprised exclusively 2024 school-going children from urban and rural populations. Table 1 shows the characteristics of the school-going children of different age groups involved in the study. The mean age of the children was 10.92 ± 2.73 years, and 886 (44%) children were below 10 years of age. Females (n =1031) were more in number than males (n =993).

**Table 1:** Comparison of demographic characteristics between urban and rural populations

Parameters	Study Groups			p-Value
	Rural, n (%)		Urban, n (%)	
Age group (in years)				
6-7	229 (11.12%)		226 (10.97%)	0.963
8-9	216 (10.72%)		215 (10.68%)	0.963
10-11	185 (9.19%)		195 (9.68%)	0.963
12-13	198 (9.83%)		192 (9.53%)	0.963
14-15	184 (9.14%)		184 (9.14%)	0.963
Gender				
Male	499 (24.65%)	494 (24.40%)		0.824
Female	513 (25.34%)	518 (25.59%)		0.824

The average prevalence of refractive error was 17.43% (Table 2). It was higher in urban areas (22.14%) than in rural areas (12.71%). The difference in prevalence was statistically significant (p-value <0.05).

**Table 2:** Distribution of study population according to the presence of refractive errors

Refractive Error	Rural, n (%)	Urban, n (%)	p-Value
Absent	884 (43.64%)	789 (38.93%)	<0.0001
Present	128 (6.36%)	223 (11.07%)	<0.0001

The maximum prevalence of refractive error was found in the age group of 6-7 years (Table 3). It was more prevalent in urban areas compared to rural areas in all age groups.

**Table 3:** Age and gender-wise distribution of the study population with refractive errors

Parameters	Study Groups			p-Value
	Rural, n (%)		Urban, n (%)	
Age group (in years)				
6-7	47 (13.39%)		68 (19.37%)	0.217
8-9	33 (9.40%)		57 (16.24%)	0.217
10-11	6 (1.71%)		12 (3.42%)	0.217
12-13	12 (3.42%)		26 (7.41%)	0.217
14-15	30 (8.55 %)		60 (17.09%)	0.217
Gender				
Male	67 (19.09%)	108 (30.77%)		0.480
Female	61 (17.38%)	115 (32.76%)		0.480

A total of 133 (37.8%) children had ocular complaints with refractive error (Table 4). The most common ocular complaints were blurring and eyeache.

**Table 4:** Distribution of the study population with refractive errors according to common complaints

Complaints	Rural	Urban	p-Value
Blurring	25 (6.55%)	41 (10.83%)	0.743
Eyeache	28 (7.13%)	35 (9.40%)	0.743
Headache	1 (0.28%)	1 (0.28%)	0.743
Watering	1 (0.28%)	1 (0.28%)	0.743

Myopia was the most common refractive error present in the school-going children, followed by hypermetropia (Table 5).

**Table 5:** Distribution of the study population according to the type of refractive error

Types of Refractive Error	Rural, n (%)	Urban, n (%)	p-Value
Simple myopia	90 (24.22%)	153 (42.17%)	0.745
Simple hypermetropia	20 (5.70%)	40 (11.40%)	0.745
Compound myopic astigmatism	12 (3.42%)	13 (3.70%)	0.745
Compund hypermetropic astigmatism	2 (0.57%)	2 (0.57%)	0.745
Simple myopic astigmatism	8 (2.28%)	19 (5.41%)	0.745
Mixed astigmatism	1 (0.28%)	1 (0.28%)	0.745

## Discussion

Refractive errors affect most of the population. They are of particular concern to children, especially school-going children. The visual system in children is still in the developing phase and any cause of a diminution of vision can lead to amblyopia. Hence, children with undetected refractive error are at risk of developing anisometric amblyopia [13].

In the present study, as shown in Table 1, children aged 6-15 years were included, with a mean age of 10.92±2.73 years. The rural and urban populations were comparable concerning mean age, as the difference was statistically insignificant. It was found that the distribution of females (n=1031; 50.93%) was higher than that of males (n=993; 49.06%). The rural and urban populations were comparable concerning gender, as the difference was statistically insignificant.

As shown in Tables 2 and 3, it was observed that the overall prevalence of refractive errors was 17.43%. The maximum refractive error was found in the age group of 6-7 years. A total of 181 (51.56%) children were newly diagnosed with our screening. The prevalence of uncorrected refractive error, especially myopia, was significantly higher in 6-15-year-old school children from urban areas (22.14%) when compared to children from rural schools (12.71%), and the difference was statistically significant (<0.0001).

In a study by Uzma et al., it was found that the overall prevalence of refractive errors was 17.6% [14]. This was similar to the present study. They also found that the prevalence of ametropia was 25.7% in urban areas and 8% in rural areas. Thus, prevalence was higher in urban areas, as seen in the present study.

In the present study, as shown in Table 4, it was found that among the cases having a refractive error, most of the cases did not have any presenting complaint. Blurring (n=66; 17.38%) and eyeache (n=63; 16.53%) were the most common complaints, whereas headache (n=2; 0.56%) and watering (n=2; 0.56%) were the least common complaints. The spectrum and distribution of the complaints were similar in rural and urban areas, as the difference was statistically insignificant. In a cross-sectional study to assess the prevalence of visual impairment in rural and urban school-going children by Prabhu et al., they noted that watering was the most common complaint (6.7% of all the students), followed by blurring or difficulty seeing the blackboard (5.4%) [15]. The spectrum was almost similar to the present study.

Also, amongst the cases having a refractive error, it was found that simple myopia was the most common refractive error, (n=243; 66.39%), followed by simple hypermetropia (n=60; 17.10%), simple myopic astigmatism (n=27; 7.69%), and compound myopic astigmatism (n=25; 7.12%). Compound and hypermetropic astigmatism and mixed astigmatism were the least common refractive errors (n=6), comprising less than 2%. The spectrum and distribution of the refractive errors were similar in rural and urban areas, as evidenced by the statistically insignificant difference. In a study conducted by Khandekar et al., they found that myopia was the main type of refractive error, comprising 57.02% of the affected population [16]. Hyperopia comprised 17.44% of the affected population, and astigmatism was the least common (5.1%) of the affected population. The distribution pattern of refractive error was similarly observed in the present study.

Nowadays, with the increase in the use of electronic gadgets and mobile phones, and the implications of online classes, the prevalence of refractive errors has increased. Our study also shows that the prevalence of refractive error is higher in children in urban areas as compared to children in rural areas, pointing towards the possible ill effects of excessive use of electronic gadgets among children and the need to encourage them to play outdoor games.

Limitations

Our study has limitations in terms of a small sample size and a localized area of study as it conducted in a particular zone in north India. As India comprises different races of people living in different parts of the country, a study with a bigger sample size done in a larger area would have been more informative.

## Conclusions

Our study shows that the most common refractive error in the age group of 6-15 years is myopia. Our study also underlines the importance of regular screening of children at schools, as undetected refractive errors among children can lead to amblyopia. As this study was conducted in a particular area, a population survey of the prevalence of refractive errors in school-going children in different regions of India can enlighten us more on this topic.

## References

[1] Newell FW (1992). Ophthalmology- Principles and Concepts, 7th ed. Ophthalmology- Principles and Concepts.7th ed, Mosby.

[2] Dandona L, Dandona R (2008). Estimation of global visual impairment due to uncorrected refractive error [Letter to the Editor]. Bull World Health Organ.

[3] Pratt C, Bryant P (1990). Young children understanding that looking leads to knowing (so long as they are looking into a single barrel). Child Dev.

[4] Packwood EA, Cruz OA, Rychwalski PJ (1999). The psychosocial effects of amblyopia study. J AAPOS.

[5] Jose R, Sachdeva S (2009). School eye screening and the National Program for Control of Blindness. Indian Pediatr.

[6] Gilbert C, Foster A (2001). Childhood blindness in the context of VISION 2020--the right to sight. Bull World Health Organ.

[7] Baltussen R, Naus J, Limburg H (2009). Cost-effectiveness of screening and correcting refractive errors in school children in Africa, Asia, America and Europe. Health Policy.

[8] Raju P, Ramesh SV, Arvind H (2004). Prevalence of refractive errors in a rural South Indian population. Invest Ophthalmol Vis Sci.

[9] Triveni C, Divya T, Devi PR, Chowdary NL, Sirisha G (2021). Prevalence of refractive errors in school going children in rural and urban areas- a cross-sectional study. Trop J Ophthalmol Otolaryngol.

[10] Joseph E, Ck M, Kumar R (2023). Prevalence of refractive errors among school-going children in a multistate study in India. Br J Ophthalmol.

[11] Hashemi H, Fotouhi A, Yekta A, Pakzad R, Ostadimoghaddam H, Khabazkhoob M (2018). Global and regional estimates of prevalence of refractive errors: systematic review and meta-analysis. J Curr Ophthalmol.

[12] Sheeladevi S, Seelam B, Nukella PB, Borah RR, Ali R, Keay L (2019). Prevalence of refractive errors, uncorrected refractive error, and presbyopia in adults in India: a systematic review. Indian J Ophthalmol.

[13] Donahue SP (2007). Prescribing spectacles in children: a pediatric ophthalmologist's approach. Optom Vis Sci.

[14] Padhye AS, Khandekar R, Dharmadhikari S, Dole K, Gogate P, Deshpande M (2009). Prevalence of uncorrected refractive error and other eye problems among urban and rural school children. Middle East Afr J Ophthalmol.

[15] Uzma N, Kumar BS, Khaja Mohinuddin Salar BM, Zafar MA, Reddy VD (2009). A comparative clinical survey of the prevalence of refractive errors and eye diseases in urban and rural school children. Can J Ophthalmol.

[16] Prabhu AV, Ve RS, Talukdar J, Chandrasekaran V (2019). Prevalence of visual impairment in school-going children among the rural and urban setups in the Udupi district of Karnataka, India: a cross-sectional study. Oman J Ophthalmol.

